# Endovascular Treatment of Intracranial Vein and Venous Sinus Thrombosis—A Systematic Review

**DOI:** 10.3390/jcm11144215

**Published:** 2022-07-20

**Authors:** Philipp Bücke, Victoria Hellstern, Alexandru Cimpoca, José E. Cohen, Thomas Horvath, Oliver Ganslandt, Hansjörg Bäzner, Hans Henkes

**Affiliations:** 1 Department of Neurology, Inselspital, Bern University Hospital, University of Bern, 3010 Bern, Switzerland; thomas.horvath@insel.ch; 2 Neuroradiologische Klinik, Klinikum Stuttgart, 70174 Stuttgart, Germany; v.hellstern@klinikum-stuttgart.de (V.H.); a.cimpoca@klinikum-stuttgart.de (A.C.); h.henkes@klinikum-stuttgart.de (H.H.); 3 Department of Neurosurgery, Hadassah Medical Center, Hebrew University Jerusalem, Jerusalem 9103401, Israel; jcohenns@yahoo.com; 4 Neurochirurgische Klinik, Klinikum Stuttgart, 70174 Stuttgart, Germany; o.ganslandt@klinikum-stuttgart.de; 5 Neurologische Klinik, Klinikum Stuttgart, 70174 Stuttgart, Germany; h.baezner@klinikum-stuttgart.de; 6 Medical Faculty, Universität Duisburg-Essen, 45147 Essen, Germany

**Keywords:** cerebral venous sinus thrombosis, endovascular therapy, thrombolysis, thrombectomy, intracerebral hemorrhage, anticoagulation, VITT

## Abstract

Background: Cerebral venous sinus or vein thromboses (SVT) are treated with heparin followed by oral anticoagulation. Even after receiving the best medical treatment, numerous patients experience neurological deterioration, intracerebral hemorrhage or brain edema. Debate regarding whether endovascular treatment (EVT) is beneficial in such severe cases remains ongoing. This systematic review summarizes the current evidence supporting the use of EVT for SVT on the basis of case presentations, with a focus on patient selection, treatment strategies and the effects of the COVID-19 pandemic. Methods: This systemic literature review included randomized controlled trials (RCTs) and retrospective observational data analyzing five or more patients. Follow-up information (modified Rankin scale (mRS)) was required to be provided (individual patient data). Results: 21 records (*n* = 405 patients; 1 RCT, 20 observational studies) were identified. EVT was found to be feasible and safe in a highly selected patient cohort but was not associated with an increase in good functional outcomes (mRS 0–2) in RCT data. In observational data, good functional outcomes were frequently observed despite an anticipated poor prognosis. Conclusion: The current evidence does not support the routine incorporation of EVT in SVT treatment. However, in a patient cohort prone to poor prognosis, EVT might be a reasonable therapeutic option. Further studies determining the patients at risk, choice of methods and devices, and timing of treatment initiation are warranted.

## 1. Introduction

**Figure 1:**
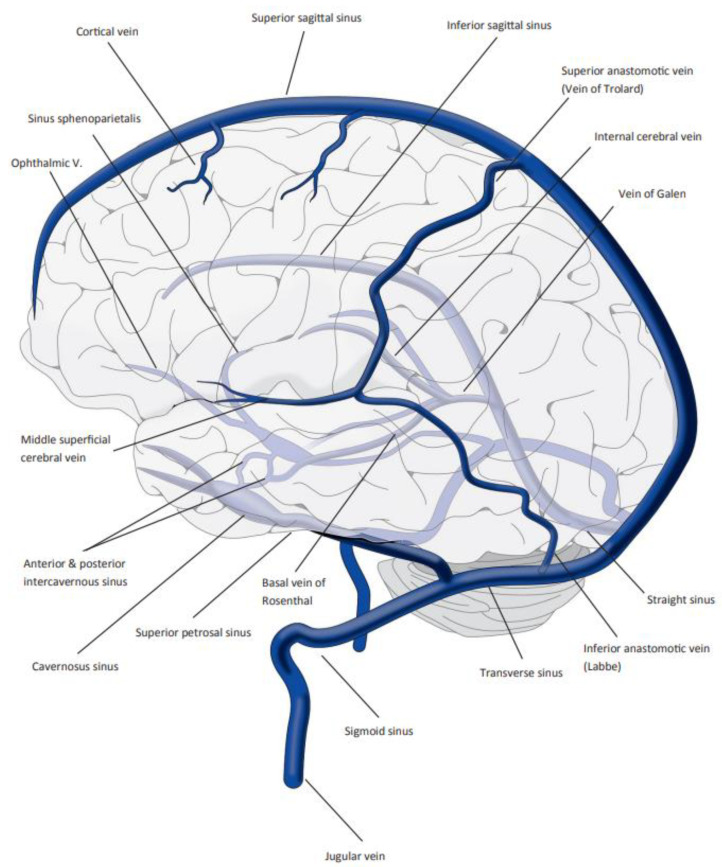
Illustration of the anatomy of the cerebral sinus and veins [7].

Cerebral venous sinus or venous thrombosis (SVT) is a rare but potentially severe cause of cerebral hemorrhage or cerebral venous infarction (incidence: 1.32 per 100,000 person-years (2.78 per 100,000 person-years in women 31–50 years of age)) accounting for approximately 0.5% of all stroke cases [1,2,3]. Various factors are associated with the development of SVT [1,4]. Recent publications have reported a considerably higher incidence of SVT in the COVID-19 pandemic because both COVID-19 (reported incidence: 8.8 per 10,000 infections over 3 months) and COVID-19 vaccination (leading to vaccine-induced immune thrombotic thrombocytopenia (VITT)) appear to increase the risk of SVT [5,6]. Figure 1 illustrates the anatomy of the cerebral dural sinus and the deep cerebral veins.

Outcomes after SVT in general are favorable assuming early diagnosis and treatment initiation [8]. Treatment is challenging, and its success highly depends on rapid anticoagulation with unfractionated or low-molecular-weight heparin, even in cases of intracerebral hemorrhage [9,10]. However, probably because of a limited capacity to dissolve an extensive intravenous thrombus load, many patients show deterioration, and as many as 13% eventually die or remain severely disabled despite sufficient anticoagulation therapy [11,12,13]. Patients with coma or altered mental status, intracerebral hemorrhage (ICH), underlying malignancy or an infection of the central nervous system appear to be at risk [12]. Pregnant or postpartum women, chronic hypertension as well as superior sagittal sinus and cortical vein involvement seem to be associated with ICH complications in SVT [14].

Endovascular treatment (EVT) strategies have been proposed to increase the frequency of good functional outcomes in high-risk or deteriorating patients [15,16,17,18,19,20,21,22,23,24,25]. Initially, endovascular thrombolysis with application of the thrombolytic agent locally and at the site of the occluded sinus was described [15,16]. Occasionally, the catheter is left in situ for 24 h or more [16,17]. In addition, several endovascular techniques have been discussed and investigated: rheolytic catheter thrombectomy, direct aspiration thrombectomy, balloon-guided thrombectomy or angioplasty and stent retriever thrombectomy [18,19,20,21,22,23,24,25,26,27,28,29,30,31,32,33,34,35,36,37]. Information on EVT in SVT is sparse and is based primarily on case series and anecdotal data and only a single published randomized controlled trial (RCT) [19,38,39,40,41,42,43,44,45,46,47,48,49,50,51,52,53,54,55,56,57]. Therefore, the current guideline recommendations remain vague. Whereas the American Heart Association and the American Stroke Association have together stated that endovascular therapy “may be considered if deterioration occurs despite intensive anticoagulation treatment,” the European Stroke Organisation has not given any advice at all [58,59].

The goal of this systematic review is to provide an overview on the current evidence supporting EVT strategies in patients with SVT. We aimed to identify potential selection criteria for patients who might benefit from an additional EVT. Furthermore, the roles of SARS-CoV-2 infections and VITT are discussed. 

## 2. Materials and Methods

We performed a systematic literature search in the PubMed (20 May 2022) and Medline (20 May 2022) databases by using the following search terms: “sinus thrombosis AND endovascular,” “sinus thrombosis AND thrombectomy,” “[cerebral] venous thrombosis AND endovascular” and “[cerebral] venous thrombosis AND thrombectomy.” For the sub-analysis of patients with SVT caused by SARS-CoV-2 infection or after COVID-19 vaccination, additional search phrases were identified: “COVID-19 AND endovascular (AND thrombosis),” “COVID-19 AND thrombectomy (AND thrombosis),” “vaccination AND endovascular” and “vaccination AND thrombectomy.” All articles published online until 20 May 2022 were screened. Two independent raters performed the literature search (P.B. and H.H.). This review follows the Preferred Reporting Items for Systematic Reviews and Meta-Analyses (PRISMA 2020) recommendations [60].

All identified publications were required to meet the following predefined inclusion criteria: (1) RCTs or retrospective studies, case series/case reports including more than five patients 18 years of age or older; (2) reported information on functional outcomes (modified Rankin scale (mRS) after discharge (not at the time of discharge)), death and complications (e.g., symptomatic intracranial hemorrhage or procedural complications); (3) inclusion of individual patient or study data only once (screening for repeated publications including identical cases); and (4) publication in English. In the analysis of EVT in patients with COVID-19 and VITT, only articles and manuscripts evaluating endovascular procedures (and the respective indications to treat patients) were eligible. We, therefore, did not consider registry data and meta-analyses reporting the frequency and the number of interventions without mentioning procedure-specific outcome parameters. 

## 3. Results

**Figure 2:**
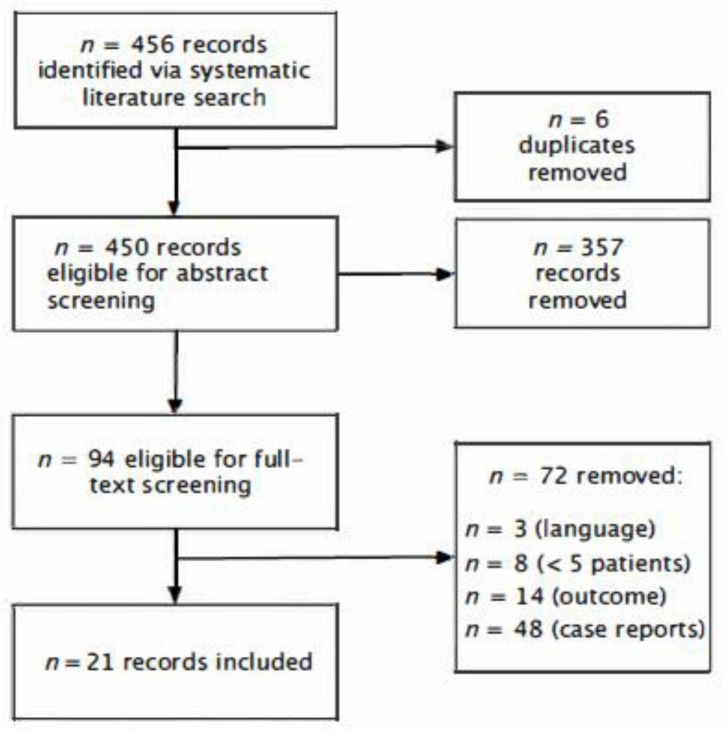
Flow diagram visualizing the selection process for the included publications.

A total of 456 records were identified and screened on the basis of the predefined search criteria (Figure 2). Only 92 were eligible for full-text evaluation. Eventually, 21 records were eligible for inclusion (reasons for exclusion of records: *n* = 56—case reports or case series with fewer than five reported events; *n* = 14—no follow-up data or data on functional outcomes; *n* = 3—not written in English).

One RCT (Thrombolysis or Anticoagulation for Cerebral Venous Thrombosis; TO-ACT) compared EVT (*n* = 33) and standard care (i.e., anticoagulation; *n* = 34) in patients with anticipated poor outcomes (Table 1) [38], which were defined as at least one of the following risk factors: mental status disorder, coma state (Glasgow coma scale (GCS) < 9), ICH or thrombosis of the deep cerebral venous system. Patients with more than 10 days from diagnosis to potential randomization, pregnancy (women in the puerperium were eligible), thrombocytopenia (platelet count < 100 × 10^9^/L), as well as clinical or radiological signs of impending trans-tentorial herniation were excluded. EVT (mechanical thrombectomy with an AngioJet (Boston Scientific, Marlborough, MA, USA), stent retriever, aspiration techniques or microcatheter) in combination with local thrombolytic treatment (urokinase administered continuously for up to 72 h) did not differ from standard care in terms of functional outcomes (mRS, 0–1 at 12 months; 67% vs. 68%; risk ratio, 0.99 (95% CI, 0.71–1.18); Table 1 provides details and information on complications).

Nyberg et al. (2017) have retrospectively analyzed their single-center cohort of patients with SVT and compared outcome data (mRS, 0–2 at 3 months) for patients treated with additional EVT (*n* = 29) to those receiving anticoagulation only (*n* = 37; 5-year time span (2011–2015)) [39]. The decision on whether to perform EVT was at the discretion of the treatment team and based on individual decision-making. The two treatment groups did not differ in outcome parameters (e.g., mRS, 0–2: 22% vs. 30%; mortality, *n* = 6 vs. *n* = 5; *p* = 1.0).

Prospective case series, retrospective analyses and case series demonstrated the technical and procedural feasibility and safety of EVT in selected patients (*n* = 19; details in Table 1) [19,40,41,42,43,44,45,46,47,48,49,50,51,52,53,54,55,56,57]. All reported patients were anticoagulated, and those with an expected poor prognosis (such as ICH or edema, neurological deterioration, coma (e.g., GCS < 9), progressive thrombus material observed on repeated imaging or signs of elevated intracranial pressure (e.g., papilledema)). Individual decision-making based on local experience and preferences was used to decide whether to perform EVT. 

A total of 25 publications met the search criteria for COVID-19 and VITT-associated EVT in SVT. Of those, 14 records were excluded (because of lack of reported patient data, review articles and registry data without information on individual patients). Eleven records were eligible for analysis (Table 2) [61,62,63,64,65,66,67,68,69,70,71], comprising case reports and case series only. Of those, four case reports and case series presented data for four patients with COVID-19 and EVT [61,62,63,64]. The decision to perform EVT was made in cases with suspected poor prognosis, with criteria comparable to those described above. Aspiration in combination with local thrombolytic therapy was the preferred endovascular technique [61,62]. The overall outcome was poor [61,62,63]. 

The first reports of SVT caused by VITT emerged in 2021 [65,66,67,68,69,70,71]. As of 20 May 2022, seven records of EVT in 16 VITT patients were identified [65,66,67,68,69,70,71]. Most cases (*n* = 15) were attributed to the ChAdOx1 nCoV-19 (AstraZeneca, Cambridge, UK) vaccine [65,66,67,69,70,71]. Only one case appeared to be associated with an mRNA vaccine (mRNA-1273 vaccine; Moderna, Cambridge, MA, USA) [68]. Excluding the latter, the time between vaccination and symptom onset was 4–27 days. Common features (except [68]) were thrombocytopenia, elevated D-dimer levels, and positivity for platelet factor 4 (PF4) antibodies (Table 2). Neurological deterioration potentially resulting in poor outcomes facilitated the use of EVT. The technical strategies described were aspiration plus stent retriever or balloon-guided thrombectomy in selected cases [65,66,67,69]. MRS of 0–1 was observed in seven patients during follow-up [65,66,69]. Three patients died [65,70].

## 4. Discussion

The overall outcome after SVT can be unfavorable in terms of functional independence and survival [8,11,72]. A total of 75–84% of patients eventually become functionally independent, with excellent functional recovery (mRS, 0–1) [8,11,72]. However, a considerable number of patients remain functionally dependent or disabled (22.2%) or die (up to 14.6%) [11,72]. To identify patients prone to poor prognosis, several risk factors have been identified (independent of treatment strategies): GCS < 9, presence of ICH, involvement of the deep cerebral venous system, mental status disorder (not specified; additional conditions such as underlying malignancy, cerebral or systemic infection, and requirement for hemicraniectomy also apply) [11,72,73,74]. Neurological deterioration—as part of the natural course of the disease or due to factors such as heparin resistance—might necessitate an additional EVT approach to remove the thrombus load [75].

Retrospective and anecdotal data have demonstrated the safety and feasibility of EVT in selected patients, and have shown satisfying results [19,39,40,41,42,43,44,45,46,47,48,49,50,51,52,53,54,55,56,57]. Yet, because of their lack of a control group and their retrospective nature, those studies have been unable to demonstrate a treatment effect resulting in an outcome benefit. In addition, inconsistencies in the timing of the interventions, indications for surgical procedures such as decompressive hemicraniectomy, etiological considerations (e.g., malignancy or sepsis) and clinical complications interfering with outcomes (e.g., status epilepticus) make an interpretation difficult. To our knowledge, only one RCT (TO-ACT) has compared EVT to standard care in patients with SVT (again, with a suspected unfavorable prognosis, as described above). TO-ACT did not detect a modification of the treatment effect attributable to EVT (see Table 1 for inclusion criteria) [38]. The limitations of this trial include its small sample size (due to early termination recommended by the data and safety monitoring board because of futility), patient selection based on clinical presentations suspected to (and previously reported to) negatively influence outcomes and unrestricted use of available endovascular approaches and devices [38]. Therefore, non-significant but clinically relevant treatment effects as well as subgroups that might actually show considerable benefits remained undetected. In a meta-analysis using individual patient data (not including RCT data), EVT was associated with poor outcomes and mortality [76]. Because each treatment decision was made individually in each case (no clear indication; suspected poor prognosis), generalizability is limited. The baseline characteristics were poorer in EVT patients than in controls. Therefore, these retrospective results must be interpreted very cautiously. Important questions regarding patient selection (clinical versus imaging characteristics) and the timing of the intervention remain unanswered. Waiting until a patient is deteriorating might be too late. 

Various treatment strategies have been investigated in EVT for SVT. Local thrombolytic therapy (urokinase or alteplase) was performed in most of the cases [19,38,39,40,41,42,44,45,46,47,48,49,51,52,53,54,55,56,57], with either a single periinterventional bolus or a continual infusion (locally, via a microcatheter) for up to 72 h (or longer, depending on the recanalization success). Yue et al. (2010) combined intraarterial thrombolysis with an endovascular thrombectomy approach (balloon-guided) [57]. Several thrombectomy procedures were discussed in the presented literature: aspiration thrombectomy [19,38,39,40,41,42,46,48,49,51,53,54,55,56], stent retriever thrombectomy [38,39,40,41,42,46,47,48,49,54], balloon-guided thrombectomy/angioplasty [19,38,39,40,41,44,47,51,53,54,57], the (rheolytic) AngioJet device [38,39,40,47,49,50] as well as additional stenting [42,46]. The study by Dashti et al. was the only one performing thrombectomy without local thrombolysis (AngioJet) [50]. A meta-analysis published in 2019 did not detect differences in outcomes stratified by treatment approach [77]. Despite poor baseline characteristics, 70–80% of patients eventually achieved mRS scores of 0–2 in the follow-up [78]. Local thrombolysis in combination with EVT has not been associated with the development or worsening of an ICH (complication rate < 10%) [78]. Further complications observed were subarachnoid hemorrhage, vessel perforation, and treatment failure [38,39,40,41,42,50,52,54]. Experience and routine (i.e., adherence to a local protocol) might be important because centers following one specific approach showed higher recanalization rates (independently of the procedure or the combination of procedures chosen) than centers using various combinations of EVT strategies [77]. However, the described inconsistencies in device allocation and the small number of treated patients make comparisons regarding the superiority of any of the treatment strategies impossible. Thus, future trials are needed to make such a comparison. Endovascular trials in acute ischemic stroke have indicated that devices are crucial in facilitating treatment effects, recanalization rates, and good functional outcomes [79].

Both COVID-19 and COVID-19 vaccination have been reported to be associated with an increase in SVT incidence [5,6]. Patients with COVID-associated SVT (4.2% of all COVID-associated strokes) appear to be older, do not have specific risk factors and experience higher in-hospital mortality (up to 16.7%) [80,81]. In a New York cohort study, two of the 12 patients received endovascular local thrombolysis [5]. SVT associated with COVID-19 vaccination may be more severe and has higher reported mortality rates (39.2% as compared with approximately 2–5% in pre-pandemic SVT) [6]. The mechanism of pathogenesis (VITT; a prothrombotic state associated with an immunoglobin G reaction against PF4) predominantly occurs in adenovirus vector-type vaccinations (ChAdOx1 nCov-19 (AstraZeneca); recombinant adenovirus type 26 vector encoding S glycoprotein of SARS-CoV-2 (Johnson and Johnson/Janssen)) [6,82]. EVT might be reasonable in selected patients meeting the TO-ACT inclusion criteria (Table 1) [38,83]. Although observational data and case reports of EVT in those patients are scarce [65,66,67,68,69,70,71], these studies have indicated the feasibility and safety of EVT and have suggested that EVT can achieve good functional outcomes in a cohort with overall poor prognosis. 

This systematic review is limited by the quality (and sample size) of the available data. The disadvantages of the TO-ACT trial have already been discussed. Because of the retrospective design of the remaining data, all the attributed limitations apply (e.g., selection bias or bias by indication) because the rationale for treatment allocation and the number of patients who were potentially eligible for treatment but were not considered are unknown. Therefore, the presented results and the conclusion require very cautious interpretation. 

In conclusion, the available data do not support the routine use of EVT strategies in patients with SVT. In patients with a suspected poor outcome (meeting the TO-ACT inclusion criteria), EVT can be performed as part of individual healing attempts. EVT is feasible and safe and might possibly improve functional outcomes. No reasonable recommendation can be made regarding which endovascular technique to use (and in which cases). According to our own experience, patients with substantial thrombus material (without an early response to anticoagulation), those with ICH, those in need of intensive care, and those with VITT do benefit from EVT. Yet, whether patients with clinical and/or imaging risk factors might benefit from early treatment initiation (before deterioration occurs) remains unknown. Further RCTs are warranted to investigate treatment strategies, patient selection, and the timing of the intervention (using predefined therapeutic strategies and reproducible inclusion criteria).

## Figures and Tables

**Table 1:** Summary of the 21 records included in this systematic review.

Reference				Treatment				Outcome Assessment				Localization					Complications		
Author (Year)	Study Type	Period	*n* (N)	Inclusion Criteria/Treatment Allocation	Pre-ICH	Endovascular Treatment	Control Group	FU (Month)	Outcome	Death	Recanal.	SSS	SS	Sig S	TS	DV	Catheter *	ICH **	Other
Coutinho et al. (2020) [38]	RCT	2011–2016	33 (67)	High probability of poor outcome, at least 1 of the following risk factors: mental status disorder, coma state (GCS <9), ICH, or thrombosis of the deep cerebral venous system (exclusion: duration from diagnosis of more than 10 days, pregnancy (women in the puerperium were eligible); thrombocytopenia (platelet count, <100 × 109/L), clinical and radiological signs of impending transtentorial herniation)	*n* = 22	*n* = 33; LT (alteplase, urokinase; up to 72 h): *n* = 17 (52%); MT: *n* = 30 (91%) (AngioJet [*n* = 14], SR [*n* = 5], B [*n* = 3], aspiration (A) [*n* = 3; Penumbra], microcatheter [*n* = 3], other [*n* = 9])	*n* = 34; standard care	6, 12	mRS 0–1 (12 months): *n* = 22 (67%) vs. *n* = 23 (68%); risk ratio 0.99 (95% CI 0.71–1.18)	12 months; *n* = 4 (12%) [vs. *n* = 1 (3%)]	SSS (6–12 months): 79% vs. 52%, 1.52 (1.02–2.27); SS (6 months): 96% vs. 86%, 1.13 (0.95–1.33)	*n* = 23 (70%)	*n* = 17 (52%)	l: *n* = 12 r: *n* = 15	l: *n* = 16 r: *n* = 22	*n* = 14 (42%)	*n* = 3 (9%)	*n* = 6 (18%) (hem. compl.)	NA
Nyberg et al. (2017) [39]	retrosp	2011–2015	29 (66)	SVT (anticoagulation), decision of treatment team	*n* = 17 (*n* = 10 in CC)	LT *n* = 29 (24–72 h); *n* = 21 additional MT (A, SR, B, AngioJet or combination; not specified)	*n* = 37; standard care	3	mRS 0–2: 22 vs. 30 (*p* = 1.0)	*n* = 6 (vs. *n* = 5)	*n* = 11; full (*n* = 8), partial (*n* = 3)	*n* = 25	*n* = 0	*n* = 21	*n* = 25	*n* = 7	NN	*n* = 9	NN
Siddiqui et al. (2014) [40]	retrosp	1995–2012	63 (NN)	SVT (anticoagulation) and either coma (GCS < 9), ICH or deterioration	*n* = 29	*n* = 63; *n* = 29 LT only, *n* = 34 MT (plus LT *n* = 27; AngioJet *n* = 28, A *n* = 3, SR *n* = 1. B *n* = 2) [LT bolus and continuously depending on recanalization]	NA	3	*n* = 53; mRS 0–1: *n* = 33	*n* = 11	full (*n* = 21), partial (*n* = 18)	NN	NN	NN	NN	NN	*n* = 5	*n* = 3	NN
Guo et al. (2020) [41]	retrosp	2010–2019	56 (227 sc)	SVT under anticoagulation; ICH, lack of improvement or deterioration of symptoms	*n* = 56	*n* = 56; LT only (*n* = 41; duration: 7 days); additional MT (*n* = 15) [SR *n* = 5, A *n* = 3, B *n* = 3, combined *n* = 4)	NA	6	*n* = 54; mRS 0–2: *n* = 49	*n* = 3	(full and partial); LT *n* = 39; MT *n* = 14	NN	NN	NN	NN	NN	*n* = 0	LT *n* = 7, MTn = 1	NN
Andersen et al. (2020) [42]	retrosp	2007–2018	28 (NN)	SVT under anticoagulation; clinical deterioration and/or impaired consciousness	*n* = 18	*n* = 28; LT (*n* = 26; 12–72 h), A (*n* = 3), SR (*n* = 3), combined (more than 2 techniques; *n* = 4) incl. PTA and stenting (*n* = 2)	NA	6	mRS 0–2: *n* = 20	*n* = 5	full (*n* = 15), partial (*n* = 11), no (*n* = 2)	*n* = 16	*n* = 15	*n* = 15	*n* = 16	*n* = 7	*n* = 1 (retrop. hem.)	*n* = 8	NA
Yang et al. (2019) [43]	pros CS	2014–2018	21 (27 sc)	SVT (anticoagulation) with: ICH, mental status impairment, coma (GCS < 9), DV thrombosis, cortical venous thrombosis, intracranial hypertension, or papilledema	NN	NN	NA	12	mRS 0–2: *n* = 21	*n* = 0	full (*n* = 5), partial (*n* = 9)	*n* = 16	*n* = 0	*n* = 17	*n* = 19	*n* = 14	NN	NN	NN
Yang et al. (2021) [44]	retrosp	2017–2019	23 (NN)	SVT (anticoagulation) with: deterioration after the initiation of anticoagulation, lethargy or coma, venous infarction with hemorrhagic transformation or ICH	*n* = 8	*n* = 23; MT (B) plus LT (urokinase)	NA	6	*n* = 21; mRS 0–1: *n* = 21	*n* = 0	full (*n* = 9), partial (*n* = 13)	*n* = 20	*n* = 11	*n* = 21	*n* = 21	NN	*n* = 2 (failure)	*n* = 1	NN
Stam et al. (2008) [45]	pros CS	NN	20 (NN)	SVT (heparin) with assumed poor prognosis: altered mental status, coma, extensive edema, ICH, infarction	*n* = 14	*n* = 20, LT only (*n* = 15 additional MT [rheolytic catheter])	NA	3 (−6)	mRS 0–2: *n* = 12	*n* = 6	NN	NN	NN	NN	NN	*n* = 20	NN	*n* = 1 (ICH progress)	NN
Lu et al. (2019) [46]	retrosp	2015–2018	14 (NN)	SVT (best medical treatment); decision of treatment team	*n* = 1	*n* = 14; MT (SR or A, combination), additional stenting in *n* = 5 (in case of failure of SR or A; re- occlusion)	NA	2 (−16)	*n* = 5 (stenting); mRS 0–1: *n* = 4	*n* = 0	NN	*n* = 0	*n* = 0	*n* = 0	*n* = 5	NA	NN	*n* = 2 (increase)	NN
Qureshi et al. (2018) [47]	retrosp	2006–2011/2016/2017	14 (NN)	SVT (anticoagulation), deterioration	*n* = 7	*n* = 13 LT, MT: AngioJet *n* = 9, B *n* = 2, SR *n* = 2 (combined) [LT bolus, up to 22 h after MT in case of incomplete recanalization]	NA	1 (−3)	mRS 0–2: *n* = 10	*n* = 1	full (*n* = 3), partial (*n* = 4)	*n* = 10	*n* = 1	*n* = 10	*n* = 13	*n* = 0	NN	NN	NN
Styczen et al. (2019) [48]	retrosp	2011–2018	13 (NN)	SVT (heparin) with assumed poor prognosis: altered mental status or coma, involvement of DV, ICH	*n* = 7	*n* = 13; MT (A *n* = 4, A plus SR *n* = 9)	NA	3 (median)	mRS 0–2: *n* = 12	*n* = 1	full (*n* = 4), partial (*n* = 7)	*n* = 9	*n* = 5	*n* = 7	*n* = 10	NN	*n* = 1 (perf)	*n* = 3	NN
Mokin et al. (2015) [49]	retrosp	2010–2013	13 (NN)	SVT (plus/minus anticoagulation), decision of treatment team	NN	*n* = 13 (LT *n* = 2 [sinus or ia]; A *n* = 2; LT plus A *n* = 3; A plus SR *n* = 2; AngioJet *n* = 2, combined *n* = 3)	NA	3	*n* = 11; mRS 0–2: *n* = 5	*n* = 1	full (*n* = 5), partial (*n* = 8)	*n* = 10	*n* = 7	*n* = 0	*n* = 11	*n* = 0	NN	NN	NN
Dashti et al. (2011) [50]	retrosp	2009/2010	13 (NN)	NA; decision of treatment team	NN	*n* = 13; AngioJet	NA	NN	*n* = 9; mRS 0–1: *n* = 7	*n* = 2	full (*n* = 6), partial (*n* = 7)	*n* = 9	NN	NN	NN	NN	NN	NN	*n* = 1 (re- occl)
Lee et al. (2016) [51]	retrosp	2008–2015	10 (NN)	SVT under anticoagulation; MT in case of ICH, deep venous thrombosis, deterioration	*n* = 9	*n* = 10; MT (B plus A [combination]) plus LT (*n* = 3; before 2013; bolus)	NA	3	mRS 0–1: *n* = 8	*n* = 1	NN	*n* = 6	*n* = 3	*n* = 5	*n* = 9	NA	*n* = 0	*n* = 1	NN
Poulsen et al. (2013) [52]	retrosp	2007–2011	9 (NN)	SVT (anticoagulation), deterioration	*n* = 4	*n* = 6 MT (*n* = 5 prior LT [24–72 h]; not specified); *n* = 5 LT only	NA	6	mRS 0–2: *n* = 8	*n* = 1	full (*n* = 2), partial (*n* = 4)	*n* = 5	*n* = 4	*n* = 0	*n* = 9	*n* = 0	*n* = 0	*n* = 1 (SAH)	*n* = 3 (hem. [eVD])
Mammen et al. (2017) [53]	retrosp	(4 years)	8 (243 sc)	SVT (anticoagulation), no response or deterioration	*n* = 1	*n* = 8 (MT, A [Penumbra] plus additional B (*n* = 7) and LT (*n* = 3; bolus)	NA	6	mRS 0–2: *n* = 5	*n* = 1	full (*n* = 3), partial (*n* = 4)	*n* = 5	*n* = 5	*n* = 2	*n* = 6	*n* = 3	*n* = 0	*n* = 0	*n* = 0
Peng et al. (2021) [54]	retrosp	2017–2020	7 (NN)	SVT (anticoagulation); one risk factor (poor outcome): coma (GCS < 9), ICH, DV thrombosis	*n* = 4	*n* = 7 MT (SR; plus A *n* = 4; plus B *n* = 4; plus heparin *n* = 4, plus LT *n* = 1 [bolus])	NA	3	mRS 0–2: *n* = 6	*n* = 0	full (*n* = 4), partial (*n* = 3)	*n* = 7	*n* = 0	*n* = 4	*n* = 5	*n* = 0	*n* = 0	*n* = 0	*n* = 0
Mehdi et al. (2020) [55]	retrosp	2018/2019	7 (NN)	SVT (anticoagulation), clinical and imaging deterioration (no signs of herniation)	*n* = 3	*n* = 7 (MT; A) plus LT (*n* = 4; bolus 20 min)	NA	1 (3, 6)	mRS 0–1: *n* = 5	*n* = 0	partial (*n* = 7)	*n* = 6	*n* = 4	*n* = 0	*n* = 3	*n* = 0	*n* = 0	*n* = 0	*n* = 0
Tsang et al. (2018) [56]	CS	2014–2018	6 (NN)	SVT (anticoagulation) with deterioration or ICH	NN	*n* = 6; MT (A [Penumbra]) plus LT (urokinase)	NA	3	mRS 0–1: *n* = 5	*n* = 1	NN	*n* = 5	*n* = 2	*n* = 3	*n* = 4	NN	*n* = 0	*n* = 0	*n* = 0
Jankowitz et al. (2013) [19]	retrosp	2009–2011	6 (27 sc)	SVT (best medical treatment); clinical (progressive deficits, coma) or radiological (hem., edema) deterioration	*n* = 4	*n* = 6; MT (A) (*n* = 6), additional LT *n* = 4 (bolus)	NA	6	mRS 0–2: *n* = 4	*n* = 1	*n* = 6	*n* = 3	*n* = 2	*n* = 1	*n* = 3	NA	*n* = 0	NN	*n* = 0
Yue et al. (2010) [57]	retrosp	2005–2008	6 (28 sc)	SVT (anticoagulation) with deterioration or assumed poor prognosis: coma, altered mental state, seizure, space-occupying lesions (edema or [hemorrhagic] infarct)	*n* = 2	*n* = 6; MT (B) plus ia T (urokinase)	NA	3 (−6)	mRS 0: *n* = 5	*n* = 1	full (*n* = 5)	*n* = 6	*n* = 4	*n* = 6	*n* = 6	*n* = 20	*n* = 0	*n* = 0	*n* = 0

* catheter complications such as perforation; ** ICH; new hemorrhage or worsening of a pre-existing intracerebral hemorrhage; *n*, number of patients; N, total number of patients (patients screened: indicated by [sc]; ICH, intracerebral hemorrhage; FU, follow-up; Recanal., recanalization; SSS, sagittal superior sinus; SS, straight sinus; S Sig, sigmoid sinus; TS, transverse sinus; DV, deep cerebral veins; RCT, randomized controlled trial; restrosp, retrospective, pros, prospective; CS, case series; GCS, Glasgow Coma Scale; SVT, sinus or cerebral vein thrombosis; MT, mechanical thrombectomy; NN, unknown; NA, not applicable; LT, local (intrasinus) thrombolysis; A, aspiration thrombectomy; SR, stent retriever thrombectomy; B, balloon guided thrombectomy or angioplasty; PTA, percutaneous transluminal angioplasty; ia, intra-arterial; T, thrombolysis; mRS, modified Rankin Scale; hem, hemorrhagic or hemorrhage; retrop., retroperitoneal; compl, complication; perf, perforation; SAH, subarachnoid hemorrhage; occl, occluison.

**Table 2:** Case reports of endovascular therapy in SVT due to COVID-19 or VITT.

Reference			Etiology		Laboratory Findings	Treatment		Outcome		Location	Complications
Author (Year)	Study Type	*n*	COVID-19 (C-19), VITT	d (After Index)		Treatment Allocation	Endovascular Treatment	mRS	Recanalization		
Ostovan et al. (2021) [62]	case series	1 (of 9)	C-19	5 (?)	TP (140 T/uL), elevated D-dimer levels (>10.000 ng/mL)	ICH	LT plus MT (A)	6	full	SSS, TS	NN
Cavalcanti et al. (2020) [63]	case series	1 (of 3)	C-19	10	TP (141 T/uL), elevated D-dimer levels (>55.000 ng/mL)	edema, rapid deterioration	MT (A) plus LT (micro-catheter, cont.)	6	partial	SSS, TS, SS, DV	NN
Omari et al. (2022) [64]	case report	1	C-19	30	NN	visual deterioration, intracranial hypertension	NN	NN	NN	TS, S Sig	blindness
Sajjad et al. (2021) [65]	case report	1	C-19	20	TP (NN), PF4 antibodies (NN), elevated D-dimer levels (6.3 mg/L)	ICH plus edema, coma, deterioration	Fogarty catheter	2	full	SSS	NN
Chew et al. (2021) [66]	case series	6	VITT (ChAdOx1 nCoV-19) *	10 (−14)	TP (11 T-91 T/uL), PF4 antibodies and D-dimer levels NN	ICH (*n* = 5), progressive thrombus material, deterioration (coma)	Aspiration (Penumbra)	0–1: *n* = 3; 6: *n* = 2	satisf. (*n* = 5)	NN	*n* = 1 (ICH-progression)
Wolf et al. (2021) [67]	case series	3	VITT (ChAdOx1 nCoV-19)	4 (−17)	TP (60 T–92 T/uL), PF4 antibodies (positive), elevated D-dimer levels (2120–22,800 ng/mL)	SAH (1); ICH (2); coma due to bilateral thalamic edema (3)	MT (A [1, 3] plus B [2])	0 (1, 3); 1 (2)	full	SSS, TS (1), SSS, TS, S Sig (2)	2 MT sessions needed (2)
Cleaver at al. (2021) [68]	case series	3	VITT (ChAdOx1 nCoV-19)	8 (−27)	TP (85 T/uL [1], 23 T/uL [2], 35 T/uL [3]); PF4-antibodies positive (all), elevated D-dimer levels (15.83–30.34 μg/mL)	progr. ICH/SAH, edema and deterioration (1); progr. ICH and thrombus material (2); new ICH, status epilepticus, intubation (3)	MT (A [1], A plus SR [2])	2 (all)	full (2), partial (1, 3)	SSS (1), SS, S Sig, TS (2), SSS, S Sig, TS (3)	NN
Gurjar et al. (2022) [69]	case report	1	VITT (mRNA-1273 vaccine) **	3 months	TP (139 T/uL), PF4 antibodies (negative), elevated D-dimer levels (16.666 ng/mL)	coma, progressive symptoms	MT (not specified)	3	full	SSS, TS, S Sig	NN
Mirandola et al. (2022) [70]	case report	1	VITT (ChAdOx1 nCoV-19)	15	TP (40 T/uL), PF4 antibodies (positive), elevated D-dimer levels (18 mcg/mL)	progressive thrombus material, edema, coma and seizure requiring intubation	MT (A plus SR)	0	partial (SS), full (rest)	SSS, SS, TS, S Sig	NN
Choi et al. (2021) [71]	case report	1	VITT (ChAdOx1 nCoV-19)	12	TP (14 T/uL), PF4 antibodies (positive), elevated D-dimer levels (>32.5 mg/L [reference: < 0.5])	progressive coma	MT (not specified)	6	full	S Sig	NN
Waraich et al. (2021) [72]	case report	1	VITT (ChAdOx1 nCoV-19)	13	TP (14 T/uL), PF4 antibodies NN, elevated D-dimer levels (62.342 ng/mL)	deterioration, SAH, seizures requiring CPR	NN	NN (2 ***)	full	SSS, TS, S Sig	NN

* ChAdOx1 nCoV-19, AstraZeneca vaccine; ** mRNA-1273 vaccine, Moderna; *** not specified, mRS assumed by the authors depending on the symptom description provided; *n*, number; VITT, vaccine-induced thrombotic thrombocytopenia; d, days; mRS, modified Rankin Scale; TP, thrombocytopenia; PF4; platelet factor 4; T, thousand; ICH, intracerebral hemorrhage; SHA, subarachnoid hemorrhage; CPR, cardiopulmonary resuscitation; NN, unknown; SSS, sagittal superior sinus; SS, straight sinus; S Sig, sigmoid sinus; TS, transverse sinus; DV, deep cerebral veins; SVT, sinus or cerebral vein thrombosis; MT, mechanical thrombectomy; LT, local (intrasinus) thrombolysis; A, aspiration thrombectomy; SR, stent retriever thrombectomy; B, balloon guided thrombectomy or angioplasty; cont, continuous.

## Data Availability

There are no data beyond the analyzed publications, which became the basis for this systematic review.
